# Link to the Land and *Mino-Pimatisiwin* (Comprehensive Health) of Indigenous People Living in Urban Areas in Eastern Canada

**DOI:** 10.3390/ijerph16234782

**Published:** 2019-11-28

**Authors:** Véronique Landry, Hugo Asselin, Carole Lévesque

**Affiliations:** 1 Institut de recherche sur les forêts, Université du Québec en Abitibi-Témiscamingue, 445 Boulevard de l'Université, Rouyn-Noranda, QC J9X 5E4, Canada; veronique.landry@uqat.ca; 2 École d'études autochtones, Université du Québec en Abitibi-Témiscamingue, 445 Boulevard de l'Université, Rouyn-Noranda, QC J9X 5E4, Canada; 3 Institut National de la Recherche Scientifique, Centre Urbanisation Culture Société, 385 rue Sherbrooke Est, Montréal, QC H2X 1E3, Canada; carole.levesque@ucs.inrs.ca

**Keywords:** Aboriginal people, urban, place attachment, land, health

## Abstract

*Mino-pimatisiwin* is a comprehensive health philosophy shared by several Indigenous peoples in North America. As the link to the land is a key element of *mino-pimatisiwin*, our aim was to determine if Indigenous people living in urban areas can reach *mino-pimatisiwin*. We show that Indigenous people living in urban areas develop particular ways to maintain their link to the land, notably by embracing broader views of “land” (including urban areas) and “community” (including members of different Indigenous peoples). Access to the bush and relations with family and friends are necessary to fully experience *mino-pimatisiwin*. Culturally safe places are needed in urban areas, where knowledge and practices can be shared, contributing to identity safeguarding. There is a three-way equilibrium between bush, community, and city; and mobility between these places is key to maintaining the balance at the heart of *mino-pimatisiwin*.

## 1. Introduction

Indigenous culture, identity, knowledge, and practices are intimately linked to the land [1,2,3]. Relatedness to the land has even been said to be a determinant of Indigenous health and well-being [4]. Health of the land is thus directly related to health of the people [5,6,7,8]. Moreover, the intersection of people and land is reflected in cultural landscapes and cultural keystone places, which are tied to well-being [9,10]. 

There have been repeated governmental attempts to cut off Indigenous peoples from their lands and cultures [4,11,12]. Furthermore, an increasing proportion of Indigenous people move to urban areas, either temporarily or permanently, for various reasons, including greater access to education, work, and services [13,14,15].

Indigenous well-being depends on access to culturally safe services and places where cultural identity is understood and can be expressed with confidence [16]. Access to natural areas within cities contributes to people’s health and such places have even been called “therapeutic” [17]. Indigenous people living in cities have thus created—and benefit from—“urban indigenous therapeutic landscapes” [18]. They also attend to so-called “sacred groves”, i.e., small natural sites they protect for their contribution to cultural identity and biodiversity conservation [19,20]. Spending time in therapeutic landscapes and sacred groves allows one to maintain the link to the land, in turn having beneficial effects on self-esteem and pride to belong to an Indigenous culture [18,21]. 

In North America, Indigenous peoples belonging to the Algonquian language family use the term *mino-pimatisiwin*, which could be loosely translated as “living the good life”, to describe a state of harmony, well-being, and comprehensive health based on relationships, cultural identity, and connection to the land (Table 1). The *mino-pimatisiwin* concept is thus more encompassing than the biomedical concept of health (absence of illness). Interestingly, similar conceptions of a “good life” exist in several other areas around the world, for example, *buen vivir* or *sumak kawsay* for the Quechuas in Ecuador [22], *kametsa asaiki* for the Ashaninkas in Amazonia [23], *tjukurpa* for the Anangu people in Australia [24], *hauora* for the Māoris in New Zealand [25], and *ubuntu* in South Africa [26].

In Canada, urban areas are increasingly important in the life paths of Indigenous people [42], more than half of the Indigenous population now living in cities. Knowing that the link to the land plays a major role in Indigenous people’s health and well-being, our aim was to determine if Indigenous people living in urban areas can reach *mino-pimatisiwin*. We show that there is a three-way equilibrium between bush, community, and city; and that Indigenous people in urban areas have various ways to maintain their link to the land, notably by accessing culturally safe sites and embracing broader views of “land” (including urban areas) and “community” (including members of different Indigenous peoples). 

## 2. Materials and Methods 

This research is based on a review of the literature on *mino-pimatisiwin* and other related concepts as well as on 15 interviews with members of various Indigenous communities in Eastern Canada, recognized by their peers for their knowledge of *mino-pimatisiwin* and life in urban settings (Table 2). We first recruited the participants through purposive sampling of key informants we already knew from our networks of research collaborators, and then through snowball sampling where participants identified other key informants to include. We stopped recruiting new participants when we reached information saturation, i.e., when new interviews did not generate additional information [43]. 

Participants—10 men and 5 women belonging to different age groups—lived or had previously lived in cities for different reasons, most often related to work, education, or family. Only one participant was Atikamek; Cree participants were younger (only one ≥60 years old); Anishnaabe participants were older (none <45 years old); and women participants were younger (none ≥60 years old) and mostly Anishnaabeg (4/5). That being said, the representations of *mino-pimatisiwin* in urban settings did not differ markedly between participants. 

Semi-directed interviews—each lasting between 1 and 2 hours—allowed participants to freely express their viewpoints, while ensuring that certain themes were addressed. We based the interview guide on social representations of the land within the Kitcisakik Anishnaabeg [44]. We considered three overarching themes to explore the importance of the link to the land to the *mino-pimatisiwin* of Indigenous people living in urban areas: (i) the land, (ii) urban environments, and (iii) support from family and friends. We conducted a thematic analysis [45] with the NVivo software (Version 10, QSR International, Chadstone, Victoria, Australia).

We obtained a certificate from the Ethics Review Board of Université du Québec en Abitibi-Témiscamingue. Free, prior, and informed consent was granted by all participants, after we presented them with a form specifying the research objectives, the nature of their participation (along with the associated benefits and disadvantages), the confidentiality safeguards, and how data would be processed and disseminated. We respected the key principles of research ethics in indigenous contexts [46]. To respect the reciprocity principle, we offered a compensation to the participants in gratitude for sharing their knowledge. Following the interviews, participants had the possibility to comment on the results to ensure that their viewpoints were adequately conveyed.

## 3. Results and Discussion

### 3.1. Defining the Land

Three elements emerged from the interviews regarding how the participants defined the land. First, the land was defined either spatially, conceptually, or spiritually. Second, urban areas were often included in the definition of the land. Third, representations of the land differed between generations, reflecting diverse life paths.

The interviews revealed the multidimensional character of the social representations of the land, as already shown in [44]. The land was defined as a physical space, with or without specific geographic boundaries. Some participants referred to their community’s territory or to cultural landscapes, and explained why certain places were important [10]. Others mentioned that the land was symbolic, lacking clearly defined boundaries: “*To me, the land is not associated to a precise place*” (participant 10); “*The land is nature, it is a spiritual land*” (participant 15). 

Well-being includes both cultural and political dimensions, according to the Cree [6]. The land, however, was not evoked in a technical or political way at first, e.g., by providing a legal definition or referring to land claims. While six participants noted a political dimension to the land, they only did so late in the interviews. They referred to their role and responsibility to defend the land [47], threatened and “eaten away” by logging companies, mining companies, and outfitters. Territorial loss is indeed one of the most important factors contributing to cultural stress in Indigenous communities [4,48]. The fact that the political dimension was addressed late in the interviews can be explained by the stated purpose of the interview, which was to talk about the link to the land and its role in health and well-being. 

Many participants said the city is included within the land and that the link to the land can be maintained in urban areas: “*I always say ‘yes, I live in Val-d’Or, yes, I live in an urban area, but I’m still on Anishnaabe land’.*... *I’m ok with that, with living the* mino-pimatisiwin *in the city*” (participant 6). “*The land is where I feel good. It’s in nature, but also in town*” (participant 1). “*My perception is that I’m still on my land here* (in Val-d’Or)” (participant 2).

Older participants more frequently referred to the land as “in the bush” compared to younger adults. The word *Nopimik* used in the Anishnaabe language to refer to the bush, also means “where I am from” [49]. Representations of the land are indeed different among generations, as shown in [12,50]. Milestone events have influenced many aspects of the lifestyle, for example, treaty signature, residential schools, intensification of natural resource exploitation, and climate changes. Transformations of the land have influenced people’s lives more or less markedly depending on their age and life pathways. 

In their quest for identity, teenagers want to explore new horizons, which often entails leaving the community (and/or the bush), but only to come back later: “*As you get older,* (the land) *becomes more important. There was a time when* (my kids) *turned their back on the land, as I did myself* (when I was their age)*, but now they’re coming back*” (participant 3). Mobility (even hypermobility) between the land, the community, and the city is characteristic of Indigenous people’s lives in contemporary Canada [42,51].

### 3.2. Link to the Land in Urban Environments

Indigenous people can feel outside their traditional cultural zone in urban areas [15]: “*I suffocate in the city. All this concrete blocks my spirit*” (participant 11). “*I realize the chock one can have* (upon arriving in the city)*. In a community, if your kid is sick you can call the nurse and she’ll say ‘Perfect, I'll be there in 5 minutes’. Here in the city, you wonder where to go, who to ask?*... *In the community, when it’s 4:00 PM, it’s simple, I’m cooking supper, ‘Come on in!’ In the city, you have to plan a week ahead! It’s more complicated. It’s not easy, particularly if you’re shy or if you have low self-esteem*” (participant 3).

Participants found their own ways of nurturing their connection to the land, even when living in the city, including by creating small-scale places of cultural safety in private backyards or in public parks [15], where physical, symbolic, and spiritual relations to the land can be expressed. Feelings of security and trust are necessary to develop belonging to a place [16]. 

In the worldviews of Anishnaabe and other Algonquian peoples, all elements of the environment are alive and have a spirit, and people rely on these elements to connect with the land [29]. A participant indeed underlined the capacity of trees to cure and provide well-being, even in urban environments: “*Just by seeing or touching a tree, talking to it, you feel better*” (participant 15).

In urban areas, relationships between humans and nature occur mostly in public parks, gardens, and nature reserves [52]. Nevertheless, all participants said they travel to the bush or the community from time to time to reconnect with nature, meet with their extended family, strengthen their identity, and engage in cultural practices [15]: “*I can find balance in the city. If I go through harsh times, I take a walk in the bush. I found ways to stay connected with nature: cross-country skiing, kayak*” (participant 3).

The multidimensional character of the link to the land can be maintained in several ways, even in urban settings, as suggested in the report of the Royal Commission on Aboriginal Peoples [53]:

- Welcome visitors who live in the community or in the bush;
- Stay connected with community members with phones or social networks;
- Meet with other Indigenous people living in the city;
- Participate in cultural events (e.g., pow-wow and National Indigenous Day);
- Visit organizations and institutions providing culturally safe services;
- Defend one’s culture, people, or community;
- Cook/eat/share traditional food;
- Respect oneself, adopt a healthy lifestyle;
- Respect nature;
- Be independent;
- Engage in outdoor activities (e.g., kayak, canoe, and walk);
- Speak Indigenous languages;
- Learn/teach traditional knowledge/practices (e.g., craft, songs, medicine, and smudging).

Certain traditional practices can be remodeled in urban settings, in spaces of “cultural recovery” [15,54]; however, to lay tobacco by a tree in an urban park or to engage in traditional activities on the shore of an urban river does not provide a full equivalent to life in the bush. In other words, the mere transfer of practices from one place to another is not sufficient. 

Cultural practices take an important place in the participants’ lives, either at work or in training (some have stressed the importance of Université du Québec en Abitibi-Témiscamingue’s First People’s Pavilion in Val-d’Or). Some participants worked in organizations offering services to urban Indigenous people or defending their rights, and mentioned that it provided meaning to their presence in the city and fed their identity; it allowed them to maintain connected with the land: “*I keep my link to the land, thanks to my job with, and for my community, my people*” (participant 5). 

For most participants, it was important to be able to go in the bush occasionally, as it is a privileged place of knowledge transmission, healing, caring, and provisioning [44,55]. Young and old, women and men, have testified to the importance of the bush in their lives, for their health and well-being: “*My home, it's in the bush. Many people belonging to my generation were born in the bush.*... *Today, the bush is a place where everyone finds healing*... mino-pimatisiwin *is to be well in your mind, in your heart*” (participant 15). “*The people of Pikogan take refuge in the bush, that's where we reconnect. We need that, it's in our blood*” (participant 12).

Life in indigenous communities is not complete if it does not allow for occasional stays in the bush. The real “home” is the bush and both the community and the city are somehow “out” of home (or they are “incomplete” places). Lévesque [46] suggested that Indigenous mobility should not be framed as an opposition between community and city. The results presented here rather point to a three-way equilibrium between bush, community, and city [55]. 

The frequency and duration of stays in the bush vary from one person to another. According to the participants, health comes from the bush, which plays a role the city will never be able to play. A participant rather bluntly stated that “*Those who don’t go in the bush end up in the street*” (participant 3), echoing Christensen’s [56] finding that breakdowns in family and community, as well as detachment from cultural identity, can result in what she calls “spiritual homelessness”. As Radu et al. [40] put it: “*The constant interaction with the land, by knowing it with all five senses, guides individuals and provides what is needed to live in harmony with the environment, with each other, and with oneself. The reciprocal and dialogic relationship with nature provides not only the material needs but also the ethic, moral and spiritual underpinnings of living a good life*”.

A participant, who had returned to living in the bush after several years in his community, said he could feel his ancestors there and that he found a long-sought well-being. Several stories were shared of diabetes regulation, blood pressure stabilization, and even major improvement of children born with chronic health problems. The numerous benefits to health and well-being associated to living in the bush have been highlighted in several studies [40,57,58,59,60]. 

But going in the bush requires a right of access, a family hunting ground, and a place to stay. One must also have access to the bush, either by owning a car, knowing someone who has one, or by living close enough to go on foot or an all-terrain vehicle, snowmobile, or canoe. Workers with less flexible schedules might lack the time needed to go in the bush (long and/or frequent holidays). Access to the bush can thus be challenging for people living in urban environments, but also in communities. This is why, for example, the (Quebec) Cree School Board has instituted goose break and moose break within the curricular schedule, so that families can leave the community together to hunt. 

A participant expressed her pride of getting by in the bush, while emphasizing that similar pride can be generated by overcoming the challenges associated to adaptation to the city. She even argued that “*real nomads live in the city*” (participant 10), thereby underlining the courage, adaptability, strength, and autonomy needed to establish in the city. Maintaining one’s indigenous identity in the city through different cultural practices generates pride and nurtures self-esteem.

### 3.3. Importance of Support from Family and Friends

The family network provides support and creates a sense of solidarity among its members [50]. The family carries sharing and caring values that help maintain one’s identity and are determinants of well-being [4,30]. The interviews revealed that solidarity networks extend into urban areas; Indigenous people in the city develop a broader sense of family and community, hence adjusting the values of sharing and caring to include members of all Indigenous peoples [34,61]. The extension of solidarity networks is favored by practices requiring peer participation: caring for someone, speaking an Indigenous language, and sharing traditional food [29]. The extension of solidarity networks and the practice of collective rituals in culturally safe places underscore the importance of urban organizations, such as Native Friendship Centres, to strengthen social identity [62]. A participant said that to find a state of balance in the city, she had to go to places frequented by other Indigenous people, “*to be in touch with people like you, sharing your cultural identity, in the workplace, for example. To be in contact with your world, to practice cultural activities, but also to take your own place*” (participant 8). When their families do not live in the city, Indigenous people surround themselves with people sharing similar experiences, hence broadening their definition of community: “*When you are in an environment like Val-d'Or, the concept of people is broader: my people is Indigenous—Cree, Inuit, Innu, Anishnaabe, Atikamek*” (participant 6).

Knowledge and practices are transmitted by parents or elders, usually in the bush [55]. The participants insisted on the importance to ensure that intergenerational connections be maintained, as well as the context in which knowledge and practices are transmitted, regardless of the place of residence. When a member is away from the family network, sharing rituals provides support and creates a feeling of solidarity that is crucial to well-being. Asselin and Drainville [51] have shown that there is a cyclical dynamic between bush, community, and city, with people moving from one to another according to changes in their lives. The participants emphasized the importance of facilitating such mobility. 

## 4. Conclusions

A strong link to the land is fundamental to Indigenous people’s comprehensive health—*mino-pimatisiwin*. We show that Indigenous people living in urban areas embrace broader views of “land” (including urban areas) and “community” (including members of different Indigenous peoples). Access to the bush and relations with family and friends are necessary to fully experience *mino-pimatisiwin*. Culturally safe places are needed in urban areas, where knowledge and practices can be shared, contributing to identity safeguarding. There is a three-way equilibrium between bush, community, and city; and mobility between these places is key to maintaining the balance at the heart of *mino-pimatisiwin*.

## Figures and Tables

**Table 1:** Various spellings and meanings of the comprehensive health concept among different Algonquian peoples in Canada.

People	Spelling	Meaning	Reference(s)
**Anishnaabe**	*Mino-pimadiziwin; Minawaziwin*	Good life; well-being	[27]
	*Minawasiwin*	Well-being	[28]
	*Mno bmaadis*	Balance between all elements of life: physical, emotional, mental, and spiritual	[29]
	*Bimadiziwin*	Importance of balance for well-being	[30]
	*Bimaadiziwin*	Living a long, fruitful life in communion with family, community, other-than-human persons, the environment, the Creator, and the spirit world	[31]
	*Mino madji8in*	Well-being, harmony, and balance	[32]
	*Mnaamodzawin*	Good life	[33]
	*Minododazin*	Holistic understanding of respect reaching beyond oneself	[34]
	*Mino-bimaadiziwin*	Vision of life and philosophy	[35]
**Atikamek**	*Miro matisiwin*	-	[32]
**Cree**	*Miõo pimâtisiwin;* *Mino-pimatisiwin;* *Mino-pimatiseewin*	Good living and healthy living	[36]
	*Mino-pimatisiwin*	Relations guide good conduct, which in turn leads to *mino-pimatisiwin*	[37]
	*Miyuupimaatsiiun*	Being alive well, responsible toward the land, able to hunt and fish on the land, to pursue traditional activities, have access to good food, appreciate life, and be in relation with other community members.	[6,38,39,40]
	*Miyo-mahcihoyān*	Physical, emotional, mental, and spiritual well-being	[41]
**Innu**	*Minu inniuin*	-	[32]

**Table 2:** Participant characteristics.

ID	Gender	Age	People
**1**	Woman	18–29	Cree
**2**	Woman	18–29	Cree
**3**	Man	30–44	Cree
**4**	Woman	30–44	Cree
**5**	Man	30–44	Cree
**6**	Woman	45–59	Anishnaabe
**7**	Man	45–59	Anishnaabe
**8**	Man	45–59	Cree
**9**	Man	45–59	Anishnaabe
**10**	Woman	45–59	Cree
**11**	Man	≥60	Atikamek
**12**	Man	≥60	Anishnaabe
**13**	Man	≥60	Cree
**14**	Man	≥60	Anishnaabe
**15**	Man	≥60	Anishnaabe
